# The indirect effects of food insecurity on obesogenic environments

**DOI:** 10.3389/fpubh.2022.1052957

**Published:** 2023-01-06

**Authors:** Jeffrey Allen

**Affiliations:** Department of Health Science and Public Health, St. Bonaventure University, Allegany, NY, United States

**Keywords:** food insecurity, obesity, physical inactivity, insufficient sleep, health inequities, nutrition

## Abstract

**Introduction:**

The Centers for Disease Control and Prevention (CDC) estimates 39.8% of United States (US) residents have obesity. This study examined obesity-related factors at the county-level to determine the indirect effects on physical inactivity, insufficient sleep duration, income inequality, food insecurity, on obesity rates.

**Methodology:**

Using the 2018 Robert Wood Johnson Foundation (RWJF) County Health Rankings data set, a multiple regression analysis was conducted to measure the percentage of the obesity rate explained by physical inactivity, insufficient sleep duration, food insecurity, and income inequality *via* geographically weighted county means. RWJF combines US federal and state datasets to produce a composite dataset comprised of information primarily from adults over the age of 18 from the 3,143 counties found within US borders. The aggregate county-level data serves as the unit of measure (*N* = 3,143). The indirect relationships (the product of two direct relationships) between obesity-related variables and obesity were measured and illustrated through a path analysis model.

**Results:**

This study found the combination of independent variables explained 53% of the obesity rates in the US, *R*^2^ = 0.53, *p* < 0.001, two-tailed. This study also found that food insecurity has both a direct and indirect effect on obesity, physical inactivity, and insufficient sleep duration. Physical inactivity has a direct effect on obesity and insufficient sleep duration, along with an indirect effect on obesity. Insufficient sleep duration has a direct effect on obesity.

**Conclusion:**

This analysis found that food insecurity indirectly impacts an obesogenic environment and drives county-level BMI averages. The dataset used for analysis predates the COVID-19 pandemic but presents the effect of food insecurity during a normative year. The findings, though interesting, provide an opportunity for future research.

## Introduction

Since the 1960's, obesity rates in the United States (US) have tripled from 13% in the mid-twentieth century (1) to a national high of 39.8% based on the Centers for Disease Control and Prevention (CDC) National Health Statistics Center data brief (2). It is estimated up to one-third of the world's population (~1.46 billion) is obese (3) while two-thirds of Americans are either overweight or obese (4). The medical costs of obesity in the US were estimated to be $149.4 billion in 2014 which included the economic effect of direct costs, disability, and premature mortality (5).

Lower income adults are more likely to become obese due to working long hours, exercising less, suffering from insufficient sleep, and consuming fewer fruits and vegetables than their higher income counterparts (6). The relationship between income, physical inactivity, obesity, and poor diet (as a result of food insecurity) is not only present in minority-majority urban communities; it is found in majority white rural populations, as well. Low income minority populations are more likely to live near unhealthy food retailers increasing the possibility of consuming a poor diet. Understanding the food environment may provide avenues to reducing obesity in these areas. High levels of obesity were validated in areas with limited access to full-service supermarkets in food deserts (7). Food swamps, areas with high concentrations of junk food retailers, and food deserts appear in communities with higher levels of income inequality and obesity (8). Quick service restaurants and discount retail outlets in “food deserts”, those economically depressed communities without access to a supermarket or healthy food outlet, serve as food outlets in lower income communities (9). The current literature involving the effect of food insecurity on obesity does not consider its effect on a combination of obesity-related community variables. Either food insecurity does or does not affect obesity directly but its effect is negligible in the studies examined for this analysis. This study primarily seeks to determine the direct and indirect effects food insecurity has on a community of variables commonly found in obesogenic environments.

CDC recognizes that the current community-level obesity reduction strategies should include additional variables and approaches to begin reducing the US obesity rate (10). RWJF has also recognized that broadening the social system variables contributing to obesogenic environments in research has the potential to more effectively develop community-level strategies to combat a variety of health disparities, including obesity. An obesogenic environment has been described as the “the sum of influences that the surroundings, opportunities, or conditions of life have on promoting obesity in individuals or populations (11).”

Physical inactivity (12–14), insufficient sleep duration (15), food insecurity (16, 17), and income inequality (18, 19) have each been associated with obesity in the literature. Based on an ecological model of obesity, this study will employ multiple regression and path analysis to examine these obesity-related factors in individuals at the county-level to determine the effects of physical inactivity, insufficient sleep duration, income inequality, and food insecurity on US obesity rates, based on available data from the 2018 County Health Rankings and Roadmaps dataset. The primary focus of this study is to determine the direct and indirect effects of food insecurity on obesity rates. In addition, the results of the path analysis will reveal the estimated direct, indirect (the product of two direct effects), and synergistic effects of food insecurity and the additional independent variables on obesity county-level obesity rates. Further, existing efforts and suggestions to blunt factors found within the community environment that can create an obesogenic environment will be discussed.

### Research questions

1. To what extent does the combination of insufficient sleep duration, physical inactivity, food insecurity, and income inequality influence obesity rates in the US?
2. In an obesogenic environment that includes insufficient sleep duration, physical inactivity, and levels of income inequality, what are the direct and indirect effects (the product of two direct effects) of food insecurity on obesity rates?

## Methodology

### Participants

This study employed the Robert Wood Johnson Foundation (RWJF) County Health Rankings and Roadmaps data set for the 2018 calendar year. Rather than surveying smaller participant populations through a primary data survey instrument, the RWJF County Health Rankings and Roadmaps data set is comprised of current data from more than 20 large federal and state cross-sectional surveys to provide geographically-weighted county averages offering an opportunity to measure the relationships among obesity and obesity-related aggravators on a national level (20). Obesity-related aggravators reviewed include physical inactivity, food insecurity, income inequality, and insufficient sleep duration, since these aggravators exist in nearly every county in the US. While the relationship between insufficient sleep and obesity is the subject of more current research, a noteworthy amount of US obesity research has focused on physical inactivity and obesity amongst the working poor. Food insecurity has been associated with obesity and the remaining independent variables separately but a cursory review of the literature did not reveal any prior research that included this combination of variables or considered the indirect effects on obesity created by the coexistence of these variables at the county-level. The unit of analysis for this study was 3,143 US counties, which is every county found within US borders. Per their terms of use, the data is available to use for personal, informational or non-commercial purposes (20).

### Variables

Adult obesity data are the age-adjusted, county-level average derived from the CDC Diabetes Interactive Atlas. BMI calculated from the height and weight proportions reported by respondents 20 years of age or older (20, 21). BMI for less populated counties reflects a several year average for the weighted measure. Physical inactivity data are the county-level average of adults 20 years of age or older reporting no leisure-time physical activity during CDC Diabetes Interactive Atlas interviews (20, 22). Insufficient sleep is the 2016 Behavioral Risk Factor Surveillance System (BRFFS) self-reported county-level average of adults 18 years of age or older reporting less than seven hours of sleep per night (20, 23). Food insecurity is the county-level percent average of respondents 13 years of age or older who reported not having a reliable source of food during the last year. The information was originally compiled by Map the Meal Gap with 2015 data from the Census Population Survey (CPS), American Community Survey (ACS), and the Bureau of Labor Statistics (BLS) and made available at the county-level (20, 24). Income inequality data represents the self-reported household income averages between 2012–2016 collected by the US Census Bureau to produce the ACS 5-year estimates (20, 25).

### Research design

The publicly-available secondary data set was downloaded over the internet from the RWJF County Health Rankings and Roadmaps website then stored as a Microsoft Excel spreadsheet on a password-protected personal computer. The variables included in this study were copied from the original Excel file to a second study-specific Excel spreadsheet for analysis. The reduced Excel spreadsheet was then exported to SPSS for data analysis *via* correlation analysis and multiple regression. The RWJF data set was then entered into SPSS AMOS to produce a path analysis model.

### Data reliability/quality assurance

The reliability of the primary data used is one of the concerns when estimating values for relatively small areas like counties. Users should be aware that reliability can vary by place and by measure. An easy estimate of reliability is the error margin for a measure. Larger error margins suggest lower reliability (20). To forestall the issue of reliability, the measures used in this study had a CI of 95%. “Although the reliability of some Couthy Health Rankings' measures varies, when multiple measures are used to capture an underlying concept, reliability improves” (20). The food insecurity variable is comprised of multiple measures to improve reliability.

### Data analysis

The variables were first reviewed *via* correlation analysis to determine significant relationships and preclude any concerns regarding multicollinearity. The variables exhibiting significant correlation with obesity were placed in the multiple regression matrix to determine the collective effect of physical inactivity, food insecurity, insufficient sleep, and income inequality on county-level obesity rates. In addition, the assumptions of multiple regression were validated. Path analysis can measure the *R*^2^ found in multiple regression but does not contain the ability to examine whether the assumptions of regression, linearity, multivariate normality, homoskedasticity, no multicollinearity, and an independence of errors, were met. The RWJF data set was then imported into SPSS AMOS to illustrate both the direct effects among the independent and dependent variables and their indirect effects on obesity relationships to provide a window into future research. Path analysis is an extension of multiple regression, which utilizes the standardized beta weights found in a multiple regression output (26). Path analysis is measured through direct, indirect, and cumulative effects the independent variables have on obesity. Path analysis modeling allows the reader to quantify and envision the indirect effects of each variable on obesity. The visualization of relationships may provide avenues of further research into the indirect effects of food insecurity.

## Results

The first research question seeks to examine the influence among the combination of physical inactivity, insufficient sleep duration, food insecurity, and income inequality on the variance in obesity rates in the US. The correlation analysis reveals that physical inactivity, insufficient sleep, food insecurity, and income inequality were all significantly correlated with obesity and each other (*p* < 0.001). When physical inactivity [*r* (3,143) = 0.71, *p* < 0.001, two-tailed], insufficient sleep duration [*r* (3,143) = 0.46, *p* < 0.001, two-tailed], food insecurity [*r* (3,143) = 0.40, *p* < 0.001, two-tailed], and income inequality [*r* (3,143) = 0.15, *p* < 0.001, two-tailed] increase, obesity rates rise. Following the correlation examination, a multiple regression model (Table 1) was examined to resolve the first research question and to establish whether these variables produced a good fit for path analysis modeling.

**Table 1:** Regression analysis summary for variables predicting obesity.

**Variable**	***B***	**^+^SEB**	**β**
Physical inactivity	0.53	0.01	0.61^**^
Food insecurity	0.13	0.02	0.11^**^
Insufficient sleep	0.17	0.02	0.15^**^
Income inequality	−0.07	0.00	−0.11^**^

*N* = 3143. B is the unstandardized coefficient.

^+^Standard Error of B.

β is the standardized coefficient.

^**^p < 0.001.

The regression model predicting obesity rates from physical inactivity, insufficient sleep duration, food insecurity, and income inequality was significant, *F* (4, 3,139) = 884.90, *p* < 0.001, two-tailed. Specifically, 53% of the variance in obesity was explained by model; when adjusted for sample size and number of predictors (independent variables), the amount of variance remained unchanged at 53%. For every percent increase in physical inactivity, food insecurity, insufficient sleep duration, and increases obesity rate by 0.53, 0.13, 0.17%, respectively. Every percent increase in income inequality reduces obesity by 0.07%.

**Figure 1:**
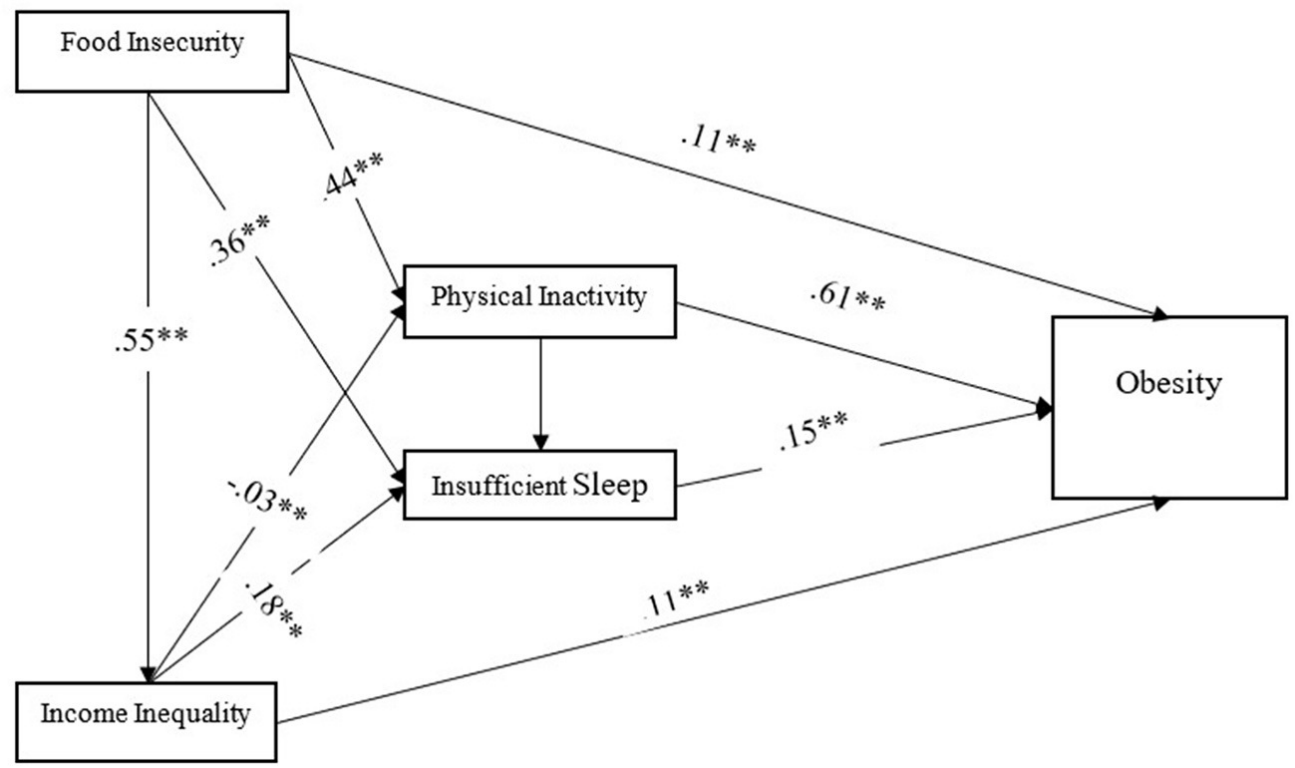
Path analysis model. *N* = 3143. ^**^p < 0.001, two-tailed.

To satisfy the remaining research question, a path analysis model (Figure 1) was created to illustrate and explain the direct (Table 2) and indirect (Table 3) effects the independent variables have on obesity rates. Table 2 displays the direct effects for each variable. This model has a good fit, NFI = 0.91, IFI = 1.0, and CFI = 1.0. Figure 1 indicates food insecurity has a direct effect on obesity (β = 0.11), a direct effect on physical inactivity (β = 0.44), and a direct effect on insufficient sleep duration (β = 0.36). Physical inactivity has a direct effect on obesity (β = 0.61) and a direct effect on insufficient sleep duration (β = 0.28). Insufficient sleep duration has a direct effect on obesity (β = 0.15). Income inequality has a direct effect on obesity (β = −0.11), a direct effect on insufficient sleep (β = 0.18), and a direct effect on food insecurity (β = 0.55).

**Table 2:** Path analysis direct effects.

**Variable**	**Physical inactivity**	**Insufficient sleep**	**Food insecurity**	**Income inequality**	**Obesity**
Physical inactivity	–	–	–	–	–
Insufficient sleep duration	0.28^**^	–	–	–	–
Food insecurity	0.44^**^	0.36^**^	–	–	–
Income inequality	−0.03^**^	0.18^**^	0.55^**^	–	–
Obesity	0.61^**^	0.15^**^	0.11^**^	−0.11^**^	–

^**^p < 0.001, two-tailed.

**Table 3:** Path analysis indirect effects.

**Variable**	**Physical inactivity**	**Insufficient sleep**	**Food insecurity**	**Income inequality**	**Obesity**
Physical inactivity	–	–	–	–	–
Insufficient sleep duration	–	–	–	–	–
Food insecurity	−0.02^**^	0.22^**^	–	–	–
Income inequality	–	0.01^**^	–	–	–
Obesity	0.04^**^	–	0.29^**^	0.01^**^	–

^**^p < 0.001, two-tailed.

Figure 1 displays only direct effects. Indirect effects are calculated by multiplying two direct effects. Table 3 indicates food insecurity has an indirect effect on obesity (β = 0.29), an indirect effect on physical inactivity (β = −0.02), and an indirect effect on insufficient sleep duration (β = 0.22). Physical inactivity has an indirect effect on obesity (β = 0.04). The variable with the most outsized indirect influence on obesity in this obesogenic environment is food insecurity.

Physical inactivity remains a primary predictor of obesity within this environment of variables but the indirect effects of physical inactivity on obesity were negligible resulting in little change in the cumulative effect of physical inactivity on obesity. Food insecurity has a small direct effect on obesity (β = 0.11) in this environment but the inclusion of indirect effects (β = 0.29) advances the influence of food insecurity to a medium-sized cumulative effect (β = 0.40) on obesity.

## Discussion

This study found that food insecurity not only directly affects community obesity rates; it indirectly affects them, as well. This study differs from the current food security literature by focusing on the indirect effect food insecurity has on common, obesity-related variables. A cursory review of the current literature did not reveal articles that appear to have connected these four phenomena to show that there appears to be causal variation. The studies reviewed for this analysis focused primarily on the impact food insecurity, as a consequence of location, has on obesity rates. Low-income households, those without adequate transportation, those in rural areas, those in food deserts, and those living in areas populated by dollar stores are often food insecure and obese (27). Additionally, rural infrastructure often does not support mass grocery deliveries, furthering limiting access to healthy foods (28). When measuring the effect of discount retailers on obesity in Kings County Washington, census tracts measures of obesity ranged from 5% in higher income communities to 30% in low-income communities. The lower-income areas included disproportionate saturation of discount retailers compared with full-service supermarkets in communities with higher mean income (16). Food environment alone, however, is not the only indicator of both the obese and food insecure. High levels obesity were validated in areas with limited access to full-service supermarkets (food deserts) but higher income neighborhood residents have higher overweight status (29). The presence of overweight suburbanites in higher income communities would suggest eating behaviors are more responsible for weight status than simply plotting supermarkets numbers and locations.

None of the studies reviewed, however, considered the indirect effect food insecurity has on other modifiable health behaviors in an obesogenic environment. Poverty seems to drive the relationships between food insecurity and both physical inactivity and sleep duration. The food insecure often rely on a combination of sources to satisfy their appetite, regardless of nutritive content. An inadequate nutrient balance can affect their ability to engage in regular physical activity and has been associated with sleep deprivation in adolescents (16).

### Implications for providers

Providers could begin to address food insecurity experienced by their patients by including specific questions regarding food security in patient intake forms, including it in a patient's history, and discuss, when applicable, during an appointment. Providing patients with budget-friendly resources, nutrition education, and support could begin to reduce the effects of food insecurity. Offering courses in budgeting, food preparation and storage, and menu planning would allow patients of limited means the knowledge essential to feel more food secure. Smartphone applications, text messaging, and internet resources can also promote nutrition education, food preparation education, and provide resources to improve nutrition in the income insecure, as well.

As an example of the resources providers could share, the Health Bucks program promoted in New York City provided nutrition education through smartphone applications, online, through live cooking demonstrations, and was available through participating food vendors throughout the city. The Health Bucks program, supported by the Healthy Food Initiative, provided a $2 match for every $5 worth of SNAP benefits used to purchase produce from neighborhood farmer's markets (20). The program provided supporting educational materials at small food vendor locations in neighborhoods considered “food deserts” (30).

### Implications for county-level public health governance

There are a variety of opportunities for county-level Public Health governance to improve food insecurity and physical activity in communities with higher levels of obesity and income inequality. Some of these options include: regulating nutritional content on restaurant menus, requiring small vendors carry a limited amount of fresh or frozen fruits and vegetables, and offering tax incentives to the owners of vacant lots to allow transient, seasonal use, venues for community gardens and farmer's markets. County or state initiatives have the opportunity to improve mass transit or ride share initiatives to reduce food insecurity in low income communities with low levels of car ownership (31). Ride share initiatives would allow low-income community members the ability to travel to full-service supermarkets. In addition, blighted vacant lots could be seized by local governments to be repurposed for community gardens, farmers markets, and school gardens. Cities which seize vacant lots for delinquent taxation have the opportunity to encourage community members to engage in collective gardening to reduce food insecurity while increasing physical activity (32).

In addition, county-level governance should encourage the participation of local schools and businesses to develop multi-component obesity remission programs. Similarly, The SHAPE UP program conducted in Somerville, MA presents another multi-component program to improve nutritional education, promote physical activity, and reduce obesity by pairing local schoolchildren with their parents to reduce the child's BMI. By involving the parent in a program designed to reduce their children's BMI, the parent would also experience a reduced BMI. In fact, parental BMI was reduced by.411 points overall (33). Through this program, education regarding food selection was printed on the menus of local restaurants, nutrition was promoted through local mass media and social media, and feedback regarding the child's progress was sent home from school with the day's healthy recipe and nutrition education of the day. This nutritional education is particularly necessary in low-income communities. Community members with less than a college degree who earn <$15,000 per year are more likely to misinterpret the nutritional value of a variety of foods or fully understand the effects of a diet replete with processed foods (34). Though these educational efforts are valuable, they should be accompanied by policy changes to improve the opportunities to reduce obesity by increasing physical activity, reducing food insecurity, and reducing income inequality at the community level.

### Limitations

This study is the product of a variety of national surveys joined at the geographic (county) level. The national surveys are the product of self-reported data and could have been improved by the use of activity diaries to catalog daily physical activity time and sleep duration. The RWJF dataset averages BMI data from several years and several sources to improve reliability but could have been improved by using objective measures of BMI exclusively rather than including self-reported height and weight. There are a variety of factors not included in this analysis which could explain the remaining 47% of the variance in obesity rates including: status and perceptions of the built environment, increased screen-time activities, perceptions and objective measures of neighborhood crime, access to exercise, insurance access and affordability, access to medical facilities, health and basic literacy, access to transportation, housing, and access to fresh food.

### Conclusions

This analysis found that food insecurity directly and indirectly impacts an obesogenic environment and may influence county-level BMI averages. The dataset used for analysis predates the COVID-19 pandemic but presents the effect of food insecurity during a normative year. The findings, though interesting, provide an opportunity for future research. Enhanced unemployment benefits during the COVID-19 pandemic reduced food insecurity by 30% and demonstrated a 42% decline in eating less (35). A comparison between the current findings and data retrieved during 2020 and/or 2021 could provide support for the indirect relationship between food insecurity and obesity.

## Data availability statement

Publicly available datasets were analyzed in this study. This data can be found here: https://www.countyhealthrankings.org/explore-health-rankings/rankings-data-documentation/national-data-documentation-2010-2019.

## Ethics statement

The studies involving human participants were reviewed and approved by Indiana State University. Written informed consent for participation was not required for this study in accordance with the national legislation and the institutional requirements.

## Author contributions

The author confirms being the sole contributor of this work and has approved it for publication.
